# Spatial and Temporal Dynamics of Hepatitis B Virus D Genotype in Europe and the Mediterranean Basin

**DOI:** 10.1371/journal.pone.0037198

**Published:** 2012-05-25

**Authors:** Gianguglielmo Zehender, Erika Ebranati, Elena Gabanelli, Renata Shkjezi, Alessia Lai, Chiara Sorrentino, Alessandra Lo Presti, Mimoza Basho, Raffaele Bruno, Elisabetta Tanzi, Silvia Bino, Massimo Ciccozzi, Massimo Galli

**Affiliations:** 1 Department of Clinical Sciences “Luigi Sacco”, Section of Infectious Diseases, University of Milan, Milano, Italy; 2 Epidemiology Unit, Department of Infectious Parasitic and Immunomediate Diseases, Istituto Superiore di Sanità, Roma, Italy; 3 Department for Control and Prevention of Infectious Diseases, Public Health Institute, Tirana, Albania; 4 Division of Infectious and Tropical Diseases, IRCCS San Matteo Hospital, University of Pavia, Pavia, Italy; 5 Department of Public Health, Microbiology and Virology, University of Milan, Milano, Italy; 6 Control of Infectious Diseases Department, National Institute of Health, Tirana, Albania; University of Florida, United States of America

## Abstract

Hepatitis B virus genotype D can be found in many parts of the world and is the most prevalent strain in south-eastern Europe, the Mediterranean Basin, the Middle East, and the Indian sub-continent. The epidemiological history of the D genotype and its subgenotypes is still obscure because of the scarcity of appropriate studies. We retrieved from public databases a total of 312 gene P sequences of HBV genotype D isolated in various countries throughout the world, and reconstructed the spatio-temporal evolutionary dynamics of the HBV-D epidemic using a Bayesian framework.

The phylogeographical analysis showed that India had the highest posterior probability of being the location of the tree root, whereas central Asia was the most probable location of the common ancestor of subgenotypes D1–D3. HBV-D5 (identified in native Indian populations) diverged from the tree root earlier than D1–D3. The time of the most recent common ancestor (tMRCA) of the tree root was 128 years ago, which suggests that the common ancestor of the currently circulating subgenotypes existed in the second half of the XIX century. The mean tMRCA of subgenotypes D1–D3 was between the 1940s and the 1950–60s. On the basis of our phylogeographic reconstruction, it seems that HBV-D reached the Mediterranean area in the middle of the XX century by means of at least two routes: the first pathway (mainly due to the spread of subgenotype D1) crossing the Middle East and reaching north Africa and the eastern Mediterranean, and the second pathway (closely associated with D2) that crossed the former Soviet Union and reached eastern Europe and the Mediterranean through Albania. We hypothesise that the main route of dispersion of genotype D was the unsafe use of injections and drug addiction.

## Introduction

Hepatitis B virus (HBV) is an enveloped DNA virus belonging to the *Hepadnaviridae* family: its genome is a small, circular partially double-stranded DNA molecule of about 3.7 kilobases including four open reading frames (S, C, P and X) coding for seven proteins (three surface proteins, two core antigens, the polymerase and the X protein).

HBV is the leading cause of liver disease and infects an estimated 240 million people worldwide [1]. Despite the recent decrease in the rate of new cases, about 7–8,000 new diagnoses are made every year in Europe [2]. The prevalence of HBV infection in Europe varies widely: in general, it is higher in south-eastern than north-western countries [2]. The highest prevalence rates are in Turkey, Romania, Bulgaria, Greece, Albania and southern Italy [3], [4].

HBV is characterised by a high degree of genetic heterogeneity due to the use of a reverse transcriptase during viral replication. Eight main genotypes (A–H) that differ genetically by at least 8% have so far been identified [5], some of which further segregate into subgenotypes with a mean genetic distance of about 4% [6]. The genotypes and subgenotypes have a distinct ethno-geographical distribution. The ubiquitous genotypes A and D are respectively the most prevalent in north-western and south-eastern Europe/Mediterranean countries [5]. Seven main HBV-D subgenotypes have so far been described (D1–D7). Considering only Europe and the Mediterranean area, D1 is the most prevalent subgenotype in Greece, Turkey, and north Africa [7]–[10], D2 the most prevalent in north-eastern Europe (Russia, Belarus, Estonia) and Albania [11], [12], and D3 the most prevalent in Italy and Serbia [13]–[15]. Some specific D strains are restricted to more limited geographical areas: D7 in Tunisia and Morocco [16], [17], D6 in Papua and Indonesia [18], and D5 in India, where all of the main D subgenotypes are also found [19]. Finally, D4 is the dominant subgenotype in Oceania [20].

The epidemiological history of the HBV-D genotype is still unclear because of the scarcity of appropriate studies. The aim of this study was to use a recently developed Bayesian approach to virus phylogeography analysis [21] in order to reconstruct the spatial and temporal dynamics of HBV-D genotype, with particular reference to the origin and the geographic dispersion of the principal D subgenotypes circulating in south-eastern Europe, the Middle East and the Mediterranean basin.

## Methods

### HBV genotype D data set

We retrieved from public databases (Genbank at http://www.ncbi.nlm.nih.gov/genbank/) a total of 312 P gene sequences of HBV-D isolated in various countries of the world, with particular attention being given to eastern Europe, the eastern Mediterranean and the Middle East (including 63 Italian and 59 Albanian isolates previously characterised by us) [12], [22]. The viral sequences were selected on the basis of the following inclusion criteria: 1) they had been published in peer-reviewed journals; 2) there was no uncertainty about the sub-type assignment of each sequence and all were classified as non-recombinant; and 3) the city/state of origin were known and clearly stated in the original publication. The sequences were aligned using ClustalX software [23] and cropped to the same length of 990 nucleotides coding for a region of the P protein between amino acids 305 and 634, and the overlapping S, PreS2 and PreS1 regions [13]. They were manually edited using Bioedit software (Tom Hall, 2011; available at http://www.mbio.ncsu.edu/bioedit/bioedit.html). The sampling locations were Albania (n = 59), central Asia (n = 10), Egypt/Israel (n = 5), the Far East (n = 8), Greece (n = 4), India (n = 26), Iran (n = 13), Italy (n = 63), Russia (n = 53), Serbia (n = 3), South Africa (n = 6), Spain (n = 4), Tunisia (n = 41), and Turkey (n = 17).

### Likelihood mapping analysis

In order to obtain an overall impression of the phylogenetic signals in the HBV P sequences, we used likelihood mapping to analse 10,000 random quartets generated using TreePuzzle [24], [25]. A likelihood map consists of an equilateral triangle containing dots representing the likelihoods of the three possible unrooted trees for a set of four sequences (quartets) randomly selected from the data set: the dots near the corners or sides respectively represent tree-like (fully resolved phylogenies in which one tree is clearly better than the others) or network-like phylogenetic signals (three regions in which it is not possible to decide between two topologies). The central area of the map represents a star-like signal (the region in which the star tree is optimal).

### Estimated evolutionary rates and time-scaled phylogeny reconstruction

To estimate the evolutionary rates of the HBV P gene sequences, we used molecular clock models based on the isolation dates of the tips of a tree. As sampling dates were not available for all of the sequences included in the analysis, we used an external calibration approach previously adopted by other authors [26], [27]. The evolutionary rates were estimated on a smaller data set including only isolates for which the sampling years were known. The estimated mean evolutionary rate was used to define a priors probability distribution during the analysis of the main data set. The smaller data-set included 216 isolates from the same places as those of the main data set and the sampling dates ranged from 1980 to 2007. The evolutionary model that best fitted the data was selected using an information criterion implemented in JModelTest [28], which is freely available at http://darwin.uvigo.es/software/jmodeltest.html.

The evolutionary rates were estimated using a Bayesian Markov Chain Monte Carlo (MCMC) method implemented in BEAST 1.5.4 [29] under a strict and relaxed molecular clock, and an uncorrelated log normal rate distribution model. As coalescent priors, we compared three simple parametric demographic models of population growth (constant size, and exponential and logistic growth) and a piecewise-constant model, called Bayesian skyline plot (BSP). The same Bayesian MCMC method, with the same substitution, molecular clock and demographic models were used to reconstruct the time-scaled phylogeny of the main data set, and statistical support for specific clades was obtained by calculating the posterior probability of each monophyletic clade.

The MCMC chains were run for at least 50 million generations, and sampled every 5000 steps. Convergence was assessed on the basis of the effective sampling size (ESS) after a 10% burn-in using Tracer software version 1.5 ( http://tree.bio.ed.ac.uk/software/tracer/ ). Only ESS's of >250 were accepted. Uncertainty in the estimates was indicated by 95% highest posterior density (95% HPD) intervals, and the best fitting models were selected by means of a Bayes factor (BF, using marginal likelihoods) implemented in BEAST [30]. In accordance with Kass and Raftery [31], the strength of the evidence against H_0_ was evaluated as 2lnBF<2 = no evidence; 2–6 weak evidence; 6–10 strong evidence; and >10 very strong evidence. A negative 2LnBF indicated evidence in favour of H_0_. Only values of ≥10 were considered significant.

### Bayesian phylogeography

The spatial reconstruction was obtained by means of the same Bayesian framework using a continuous time Markov Chain (CTMC) implemented in BEAST [21] over discrete sampling locations. The Bayesian Stochastic Search Variable Selection (BSSVS) model was implemented, which allows the diffusion rates to be zero with a positive prior probability. Comparison of the posterior and prior probabilities of individual rates being zero provided a formal BF to test the significance of the linkages between locations. Rates yielding a BF of >6 were considered well supported and formed the migration pathway. The maximum clade credibility (MCC) tree (i.e. the tree with the largest product of posterior clade probabilities) was selected from the posterior tree distribution using the program TreeAnnotator (included in the Beast package) after a 10% burn in. The final trees were manipulated in FigTree v.1.3 for display purposes. The significant migration rates were analysed and visualised by SPREAD [32] a recently developed application available at http://www.kuleuven.be/aidslab/phylogeography/SPREAD.html.

## Results

### Likelihood mapping analysis of the data set

The phylogenetic noise of the data set was investigated by means of likelihood mapping. The evaluation of 10,000 random quartets showed that more than 92.7% of the randomly chosen quartets fell in the corners, and only 2.7% in the central area of the likelihood map, thus indicating that the alignment contained sufficient phylogenetic information (Figure S1).

### Estimated evolution rates

The mean evolutionary rate of the HBV-D P sequences was evaluated on a data-set including 216 isolates with known sampling dates.

Comparison of the strict and relaxed clock models using the BF test showed that the relaxed clock fitted the data significantly better than the strict clock (2lnBF = 316.4 in favour of the relaxed clock). Comparison of the coalescent priors showed that the BSP model was better than the constant (2lnBF = 222.27) or exponential growth model (2lnBF = 140.9).

Under the relaxed clock model, the estimated mean value of the 990-nt fragment of the P gene was 4.4×10^−4^ subs/site/year (95%HPD 2.6×10^−4^–6.2×10^−4^), and this external rate estimate was used for the subsequent analyses.

### Phylogeographical analysis

**Figure 1:**
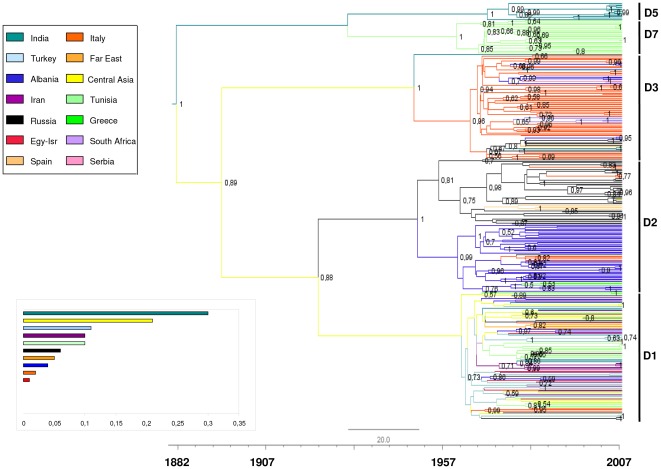
The maximum clade credibility (MCC) tree of HBV-D P gene sequences. The branches are coloured on the basis of the most probable location state of the descendent nodes (see colour codes in upper left inset). The numbers on the internal nodes represent posterior probabilities, and the scale at the bottom of the tree represents the years before the last sampling time (2007). The clades corresponding to the main HBV-D subgenotypes (D1, D2, D3, D5, D7) are highlighted.

Figure 1 shows the maximum credibility tree of the main data set, using the previously estimated mean evolutionary rate of the HBV P sequences as a prior probability distribution. The figure shows the most probable location of each branch in different colours, and the tMRCA estimates under relaxed molecular clock. Analysis of the tree showed that the 312 HBV genotype D isolates segregated into a number of significant clades corresponding to the main subgenotypes D present in the geographic area considered (D1, D2, D3, D5 and D7). In particular, HBV D5 and D7 formed two highly significant clades (pp = 1 each one) that diverged from the tree root earlier than the other subgenotypes. The isolates belonging to the three major subgenotypes (D1–D3) formed three clades (with pp values of respectively 0.73 and 1) sharing two highly significant ancestors: the first common to all three subgenotypes (pp = 0.89) and the second shared only by D1 and D2 (pp = 0.88).

Phylogeographical analysis showed that India had the highest posterior probability to be the location of the tree root (pp = 0.30), significantly higher than that of the second most probable location, the Central Asia (pp = 0.22) (see histogram in Figure 1 for more details), which was the most probable location of the deepest nodes of the tree: i.e. the common ancestors of D1–D3 and D1–D2 (pp values of respectively 0.33 and 0.43).

Two clades included isolates from homogenous geographical areas: clade D5 encompassed only Indian isolates, and D7 isolates from Tunisia (Table 1). As all of the other clades included isolates from different parts of the world, we analysed each subgenotype separately.

**Table 1:** tMRCAs and locations of the main clades.

Node	tMRCA^1^	95%HPD U^2^	95%HPD L^3^	Years	Credibility intervals	MRCA states	pp^4^
Root	128	66	210	1880	1798–1942	India	0.3
D1–D3	86	47	135	1922	1873–1961	Central Asia	0.33
D1–D2	68	37	104	1940	1904–1971	Central Asia	0.43
D1	48	27	70	1960	1938–1981	Turkey	0.40
D2	52	31	78	1956	1930–1977	Central Asia+Russia	0.63
D2A	45	27	65	1963	1943–1981	Albania	0.96
D2B	43	27	63	1965	1945–1981	Russia	0.96
D3	65	37	102	1943	1906–1971	Central Asia+India	0.56
D5	40	22	62	1968	1946–1986	India	0.95
D7	52	30	80	1956	1928–1978	Tunisia	0.99

1 tMRCA: Time of the most recent common ancestor.

2 95%HPD U 95%: Highest Posterior Density Upper.

3 95%HPD U 95%: Highest Posterior Density Lower.

4 pp: posterior probability.

**Figure 2:**
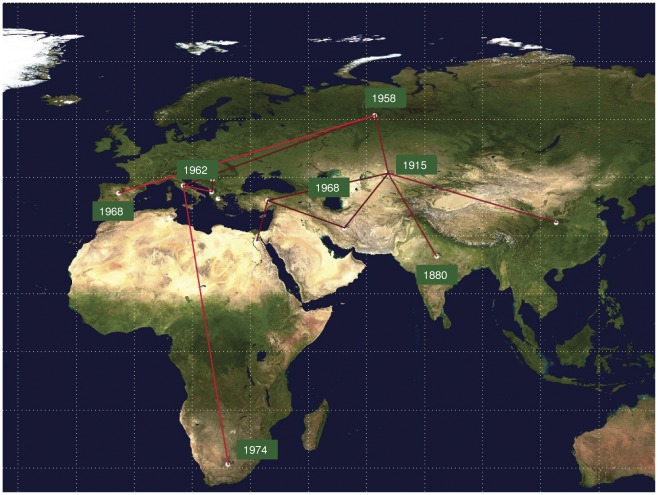
Significant non-zero HBV-D migration rates worldwide. Only the rates supported by a BF of >6 are shown. The relative strength of the support is indicated by the colour of the lines (from dark red = weak to light red = strong). The map was reconstructed using SPREAD (see Methods).

Time of the most recent common ancestor (tMRCA) estimates with credibility intervals (95%HPD) with corresponding years and most probable locations with state posterior probabilities (pp) of the main clades observed in the MCC tree of Figure 2.

The strains in clade D1 were isolated in India, Central Asia (Kazakhstan and Uzbekistan), the Far East (China and Vietnam), Tunisia, Iran, Egypt, north-eastern Europe, and Italy, and formed only small groups of no more than five isolates. Turkey had the greatest posterior probability (pp = 0.40) of being the location of the D1 MRCA, followed by Central Asia (pp = 0.30) and Iran (pp = 0.22) (Figure S2A). There was no significant geographical structure.

The clade corresponding to the D2 subgenotype (Figure S2B) included a large prevalence of isolates from Russia, Belarus and Estonia, a large group of Albanian isolates, a number of Italian strains, and sporadic isolates from Greece and Turkey. Central Asia and Russia (state pp = 0.33 and 0.30 respectively) shared the vast majority of posterior distribution with a 0.63 cumulative probability of being the location of the MRCA (followed by Albania: pp = 0.22). All of the D2 isolates tended to segregate significantly on the basis of their geographical origin. In particular, there were two highly significant sub-clades: sub-clade D2a (pp = 0.99) included all of the Albanian and several Italian strains, indicating Albania as the most probable MRCA location (pp = 0.96); the D2b sub-clade (pp = 0.81), which included all of the north-east European and a few Italian strains, had Russia as its most probable MRCA location (pp = 0.96).

The D3 clade included a number of Italian isolates, several Albanian and north-east European strains, three isolates from Serbia, four from India and one from Mongolia; a group of six South African isolates significantly segregated from the other strains. One single Indian isolate (Ne20D3) was at the outgroup (Figure S2C). Central Asia and India shared the highest posterior probability of being the location of the D3 MRCA (cumulative pp = 0.54, 0.27 for both). All of the D3 isolates tended to segregate significantly on the basis of their geographical origin.

Using a BF test under BSSVS, we identified the main linkages between the different locations. Figure 2 (see Table S1 for more details) shows all of the rates with a non-zero expectancy and a BF of ≥6. Interestingly, among the most significant rates, India linked only with central Asia (BF = 27.3), whereas central Asia was related to five locations (Russia, the Far East, Turkey, Iran and India), thus suggesting it played a central role in the global dispersion of HBV-D. Other locations with several significant linkages were Russia (with Italy, Spain, and central Asia) and Turkey (with central Asia, Iran and Egypt/Israel), and Italy (with Russia, Albania and South Africa).

### Estimated times of the most recent common ancestors (tMRCA)

Using the relaxed molecular clock model described in Methods, we estimated the tMRCA of all of the internal nodes of the Bayesian tree. In particular, the estimated mean tMRCA of the tree root was 128 years ago (95%HPD 66–210 years), thus suggesting that the origin of the D subgenotypes goes back to 1880 (credibility interval 1798–1942). HBV-D5 and D7 were the first subgenotypes to diverge from the root, although their radiation is relatively recent (40 and 52 years ago, corresponding to 1968 and 1956; see Table 1). On the basis of our estimates, the ancestor shared by D1–D3 existed a mean 86 years ago (about 1922) and that shared by D1 and D2 68 years ago (1940). Once again, the time of subgenotype radiation was more recent: 65 years ago (95%HPD 37–101) for HBV-D3 (1943), and 52 years ago (1956) for D2 and 48 years ago (1960) for D1. On the basis of the tMRCA estimates, the two D2 sub-clades (A and B) originated in 1963 (95%HPD 1943–1981) and 1965 (95%HPD 1945–1981) respectively.

## Discussion

HBV genotype D is found throughout the world: it is the most prevalent genotype in north-eastern Europe, the Mediterranean basin, northern Africa, and the Middle East; it is highly prevalent in the Indian sub-continent and a group of island in the Indian Ocean with high endemic levels of HBV (Nicobare and Andaman) [33]; and it has also been identified in Oceania [20].

It is characterised by a high degree of heterogeneity. Using a relaxed molecular clock model, we estimated the evolutionary rates of the P sequences in a sub-set of dated isolates as ranging from 2.6 to 6.2×10^−4^ subs/site/year. Previous studies have suggested evolutionary rates varying between 1.5×10^−5^ and 7.72×10^−4^ subs/site/year [34]–[39]. Recently, we showed that the average substitution rate of genotype D was higher than that of the other genotypes (particularly genotype A) and suggested that these differences were mainly due to population-related factors such as the wider spread of the HBV-D epidemic and the different main routes of transmission of the two genotypes [22]. In the last year, Harrison *et al.* [40] have reported slower viral evolutionary rates during hepatitis B **e** antigen positive state, compared to hepatitis B **e** antigen negative state. It is well known that HBV genotypes influence the HBeAg state, as a number of mutations that reduce HBeAg expression (such as those affecting nucleotide 1896 of the Pre-core region) occur more frequently in genotype D, than among others [41]. This may further explain the higher evolutionary rates of HBV-D.

On the basis of the evolutionary rate estimate obtained, we inferred the tMRCA of every internal node within the Bayesian tree of the entire data set. Interestingly, our time-scaled reconstruction supported a relatively recent history of the currently circulating HBV-D genotype with the time of the tree root being 128 years ago, in line with previous estimates [22], [39]. This estimate suggests that the common ancestor of the D genotype existed in the second half of the XIX century.

The origin of D genotype and subgenotypes and their spread throughout the world is still obscure. Ours is the first attempt to reconstruct the epidemiological history of HBV-D genotype phylodynamically and phylogeographically. Our reconstruction shows that the most probable location of the tree root is India, which suggests that the Indian sub-continent was the place in which HBV-D originated. This is supported by the fact that genotype D is highly prevalent in India, which hosts at least four HBV-D subgenotypes with very different local distributions: D1 is the most prevalent in northern India, D2 in western India, D3 is as prevalent as D2 in eastern India [42], and the recently reported D5 (the only subgenotype present in a primitive tribe in South-East India) [19]. As shown by our analysis and previous reports [43], D5 was probably the first subgenotype to diverge from the root of the phylogenetic tree.

As we were interested in studying the HBV-D subgenotypes mainly circulating in Europe and the Mediterranean area, no D4 isolates were included in our analysis. One recent study has confirmed that D5 was the first subgenotype emerging from the tree [43], and found that D4 formed a significant group with D7, a finding that is supported by a preliminary review of our own data which include a few D4 sequences (Figure S3). These observations and the very high degree of HBV-D genetic divergence suggest that India was the cradle of the HBV-D genotype.

HBV D1–D3 share more recent ancestors which, on the basis of our phylogeographical reconstruction, were most probably located in Central Asia. Our tMRCA estimate suggests that the D1–D3 MRCA existed in the first decades of the XX century (1922), with D3 being the first subgenotype to diverge in the early 1943 while the radiation of subgenotypes D1 and D2 probably occurred between the mid- 1950s and early 60s. Only a few data are available concerning HBV genotype prevalence in central Asia. HBV-D is the most prevalent genotype in Uzbekistan, Tajikistan and Kazakhstan [44]–[46], and one of these studies [11] found that D1 was the most prevalent subgenotype. Much more data support the greater prevalence of D1 than the other subgenotypes in Iran [10], [47], Turkey [8], Pakistan [48], [49] and Afghanistan [50]. In line with these data, Turkey had the highest posterior probability of being the location of origin of D1, followed by Central Asia and/or Iran. The lack of any significant geographical segregation of D1 strains suggests the relatively recent penetration of HBV-D1 in this area as a result of multiple introduction events, probably due to frequent exchanges with neighbouring countries.

HBV D2 (the most prevalent subgenotype in north-eastern Europe) has two distinct clades, one originating in Russia and the other in Albania [11], [12], [46], [51], [52]. In our reconstruction, the D2 clade as a whole probably originated somewhere between central Asia and Russia as they had similar posterior probabilities.

The phylogeography of the D3 subgenotype remains elusive although, once again, central Asia and India seem to be the most likely locations of origin. The reason for this uncertainty was the scarcity of dated HBV D3 sequences collected in places other than Italy, which represented the majority of the available strains. It has been shown that subgenotype D3 (and particular strains carrying specific mutations such as the T125M in the a determinant) is prevalent among IVDUs in various countries, including Italy, Denmark, the USA, British Columbia, Belgium and Serbia [13], [20], [53]–[56]. These observations, partially strengthened by the high prevalence of this subgenotype in India or Pakistan [49], suggest that the international drug trade may have played a role in its spread, at least in recent times. Our recent study suggested the presence of D3 in Italy even before the epidemic of intravenous drug addiction in the early 1970s, rising questions about other possible routes of geographic dispersion of this strain [22]. Nevertheless our analysis suggests that D3 was the earlier subgenotype diverging in 1940s.

Combining our temporal and spatial reconstructions of the evolutionary dynamics of HBV-D, our data suggest that, by the end of the XIX century, its ancestor left India and reached central Asia, where subgenotypes D1–D3 diverged. Central Asia was the source of their further spread to Europe and the Mediterranean area in the first decades of the XX century by means of at least two routes: a south-western route (mainly due to the diffusion of subgenotype D1) crossing the Middle East (where most probably D1 diverged) and reaching north Africa and the south-eastern Mediterranean, and a second route (closely associated with D2) towards the north-west, which crossed the former Soviet Union and reached eastern Europe and the Mediterranean through Albania [12]. A third possible epidemic of HBV in Eurasia is represented by the diffusion of D3, which still remains obscure.

The spread of the main European HBV subgenotypes dates to a period of time including the First and especially the Second World War. The importance of war in the history of hepatitis viruses has been efficiently explained by Reuben [57]. Times of war provide many opportunities for infection, such as massive vaccines administration to soldiers, wounds, blood transfusions and other violence-related factors. The largest recorded outbreak of viral hepatitis occurred in 1942, when more than 28,000 US soldiers developed jaundice (and 62 died) after being inoculated by an HBV-contaminated yellow fever vaccine [58], [59]. Subsequent studies of veterans have shown that 97% of the subjects who received this vaccine were positive for serum markers of HBV infection [60]. We can hypothesise that this or similar events, such as the outbreaks of jaundice following the intravenous injection of arsphenamine (the main anti-syphilis treatment from the early 1920s to the late 1940s) led to the spread of some HBV D subgenotypes in Europe [61], [62]. The spread of D genotype could have been subsequently amplified locally by other routes.

In particular, the rate of vertical transmission is relatively low in populations in which genotype D is predominant probably because of the high rate of mutations causing HBeAg negativity in this genotype. Consequently, the majority of infections are acquired horizontally within these populations [63], mainly as a result of the use of unsterilised needles and syringes, and household contacts [64], [65]. In line with our spatio-temporal reconstruction of HBV-D epidemics, the unsafe use of injections in medical practice was still a problem in India and the Middle East in the year 2000, when about 70% of the equipment was reused [66], and about 57% of HBV infections in India were attributable to this route of transmission [67], [68]. Unsafe injections have also been indicated as a possible cause of the spread of HBV in east European countries such as Moldavia [69] and Romania [70].

One further event that probably contributed to the rapid diffusion of HBV-D is the intravenous drug use epidemic that has affected Europe since the early 1970s. A recent literature review has pointed out that the greatest prevalence of HBsAg among IVDU populations is in the eastern Mediterranean and eastern Europe [71], where the most prevalent genotype is HBV-D.

In conclusion, HBV-D is highly prevalent in eastern and southern Europe, where HBV is highly endemic and the main transmission route is unsafe injections. On the contrary, genotype A is highly prevalent in central and northern Europe, where HBV is mainly sustained by sexual transmission [4], [65], [72]. This observation recalls the epidemiological dichotomy previously described by us in Italy [22], and suggests that similar population-related mechanisms may have played a role in the different distribution and evolutionary dynamics of the two main HBV genotypes throughout Europe.

## Supporting Information

Figure S1 **Likelihood map of the 312 HBV-D P gene sequences.** Each dot represents the likelihoods of the three possible unrooted trees per quartet randomly selected from the data set: the dots near the corners or sides respectively represent tree-like (fully resolved phylogenies in which one tree is clearly better than the others) or network-like phylogenetic signals (three regions in which it is not possible to decide between two topologies). The central area of the map represents a star-like signal (the region in which the star tree is optimal tree). The numbers indicate the percentage of dots in the centre of the triangle.
(TIF)

Figure S2 **Part of the MCC tree shown in** **Figure 1** **focusing on the D1 (a), D2 (b) and D3 (c) clades.** The branches are coloured on the basis of the most probable location state of the descendent nodes (see colour codes in upper left inset). The numbers on the internal nodes represent posterior probabilities, and the scale at the bottom of the tree represents the years before the last sampling time (2007). Subclades D2a and D2b are highlighted (panel b).
(TIF)

Figure S3 **The maximum clade credibility (MCC) tree, including also three D4 isolates.** The branches are coloured on the basis of the most probable location state of the descendent nodes (see colour codes in upper left inset). The numbers on the internal nodes represent posterior probabilities, and the scale at the bottom of the tree represents the years before the last sampling time (2007).
(TIF)

Table S1 **Significant migration rates.**
(DOC)
