# Supplementary figures and images for: Spatial and Temporal Dynamics of Hepatitis B Virus D Genotype in Europe and the Mediterranean Basin
Source: PLoS One. 2012 May 25;7(5):e37198. doi: 10.1371/journal.pone.0037198 (PMC3360700; doi:10.1371/journal.pone.0037198)

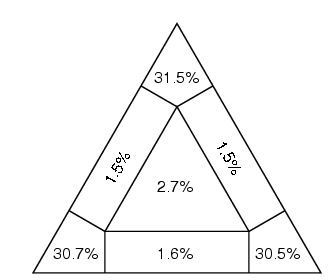

Supplement: Figure S1 — Likelihood map of the 312 HBV-D P gene sequences. Each dot represents the likelihoods of the three possible unrooted trees per quartet randomly selected from the data set: the dots near the corners or sides respectively represent tree-like (fully resolved phylogenies in which one tree is clearly better than the others) or network-like phylogenetic signals (three regions in which it is not possible to decide between two topologies). The central area of the map represents a star-like signal (the region in which the star tree is optimal tree). The numbers indicate the percentage of dots in the centre of the triangle. (TIF) [file pone.0037198.s001.tif]

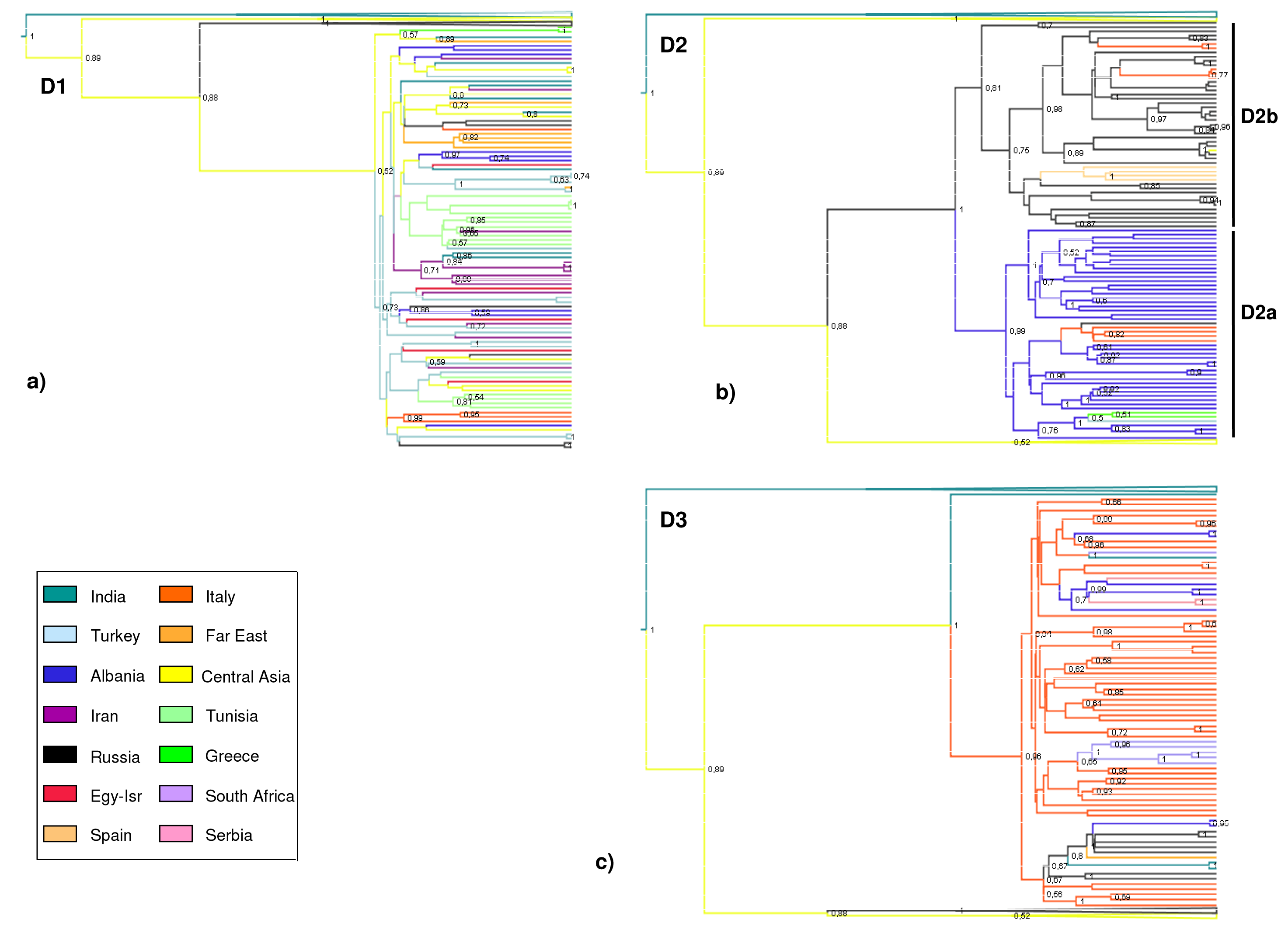

Supplement: Figure S2 — Part of the MCC tree shown in Figure 1 focusing on the D1 (a), D2 (b) and D3 (c) clades. The branches are coloured on the basis of the most probable location state of the descendent nodes (see colour codes in upper left inset). The numbers on the internal nodes represent posterior probabilities, and the scale at the bottom of the tree represents the years before the last sampling time (2007). Subclades D2a and D2b are highlighted (panel b). (TIF) [file pone.0037198.s002.tif]

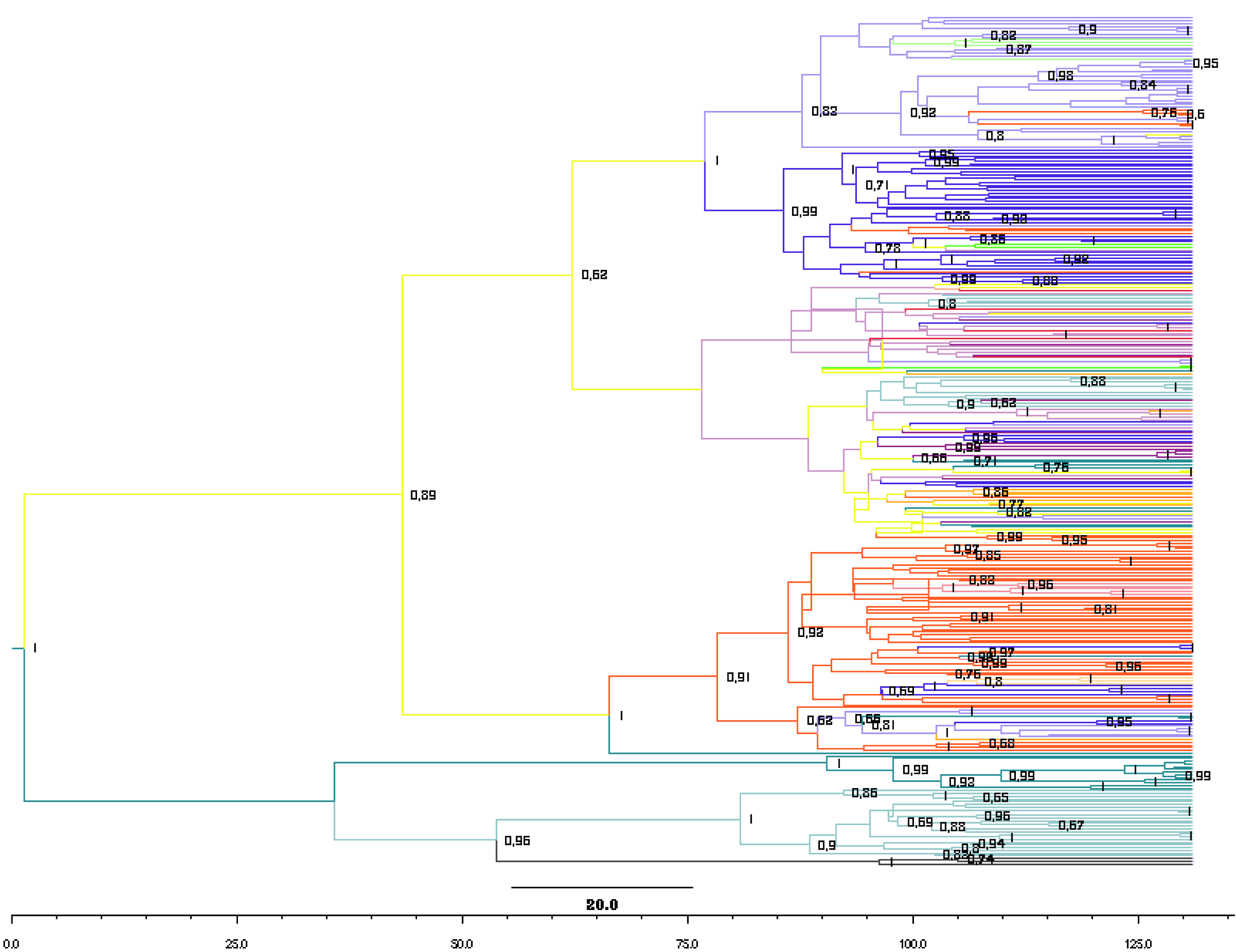

Supplement: Figure S3 — The maximum clade credibility (MCC) tree, including also three D4 isolates. The branches are coloured on the basis of the most probable location state of the descendent nodes (see colour codes in upper left inset). The numbers on the internal nodes represent posterior probabilities, and the scale at the bottom of the tree represents the years before the last sampling time (2007). (TIF) [file pone.0037198.s003.tif]
