# Supplementary material for: Spatial and Temporal Dynamics of Hepatitis B Virus D Genotype in Europe and the Mediterranean Basin
Source: PLoS One. 2012 May 25;7(5):e37198. doi: 10.1371/journal.pone.0037198 (PMC3360700; doi:10.1371/journal.pone.0037198)
Supplement: Table S1 — Significant migration rates. (DOC) [file pone.0037198.s004.doc]

**Table S1**. **Significant migration rates.**

| **Rates** | BF* |
| --- | --- |
| India-Central Asia | 27.4 |
| Central Asia-Far East | 30.4 |
| Central Asia-Turkey | 13.2 |
| Central Asia-Iran | 9.3 |
| Central Asia-Russia | 30.6 |
| Turkey-Iran | 10 |
| Turkey-Far East | 18.3 |
| Russia-Italy | 15.9 |
| Russia-Spain | 270.5 |
| Italy-South Africa | 154.1 |
| Italy-Albania | 153.6 |
| Albania-Serbia | 13.9 |

* BF: Bayes Factor
